# Supplementary material for: Diversity of Tick-Borne Pathogens in Tick Larvae Feeding on Breeding Birds in France
Source: Pathogens. 2022 Aug 20;11(8):946. doi: 10.3390/pathogens11080946 (PMC9414652; doi:10.3390/pathogens11080946)
Supplement: Supplementary file 1 [file pathogens-11-00946-s001.zip › pathogens-1870408-supplementary.pdf]

Table S1. Number of ticks per host bird species.

Tick Species / Bird Species	*I. ricinus*	*I. frontalis*	*I. arboricola*	*Ixodes* spp.	*H. concinna*	*H. punctata*	*Haemaphysalis* spp.	*Hyalomma* spp.	*Rhipicephalus* spp.	Unidentified
Acrocephalus arundinaceus	1	0	0	0	0	0	0	0	0	0
Acrocephalus palustris	35	0	0	0	0	0	0	0	0	0
Acrocephalus schoenobaenus	6	0	0	0	0	0	0	0	0	1
Acrocephalus scirpaceus	19	0	0	0	0	0	0	5	0	3
Aegithalos caudatus	1	0	0	0	0	0	0	0	0	2
Anthus trivialis	40	0	0	0	0	0	1	0	0	3
Aquila chrysaetos	0	0	0	0	0	14	0	0	0	1
Aquila fasciata	0	0	0	0	0	0	0	0	2	0
Athene noctua	0	0	1	0	0	0	0	0	0	0
Carduelis cannabina	4	0	0	0	0	0	0	0	0	0
Carduelis chloris	1	0	0	0	0	0	0	0	0	0
Cettia cetti	3	0	0	0	0	0	0	0	0	1
Coccothraustes coccothraustes	28	0	0	0	2	0	1	0	0	2
Cyanistes caeruleus	16	1	1	2	5	0	1	0	0	9
Emberiza cirlus	0	0	0	0	2	0	0	0	0	1
Emberiza schoeniclus	3	0	0	0	0	0	0	0	0	0
Erithacus rubecula	444	4	0	0	0	0	0	0	0	33
Fringilla coelebs	54	1	0	0	0	0	0	0	0	3
Garrulus glandarius	116	3	0	0	0	0	0	0	0	26
Hippolais polyglotta	13	1	0	0	0	0	0	0	0	0
Hirundo rustica	0	0	0	0	0	0	0	0	0	2
Lanius collurio	11	0	0	0	0	0	0	0	0	1
Leiothrix lutea	0	1	0	0	0	0	0	0	0	0
Limosa limosa	0	0	0	0	0	2	0	0	0	0
Lophophanes cristatus	2	0	0	0	0	0	0	0	0	0
Lullula arborea	1	0	0	0	0	0	0	0	0	0
Luscinia megarhynchos	112	0	0	0	1	0	0	0	0	9

Luscinia svecica	3	0	0	0	0	0	0	0	0	0
Motacilla cinerea	1	0	0	0	0	0	0	0	0	0
Muscicapa striata	1	0	0	0	0	0	0	0	0	1
Oriolus oriolus	1	0	0	0	0	0	0	0	0	0
Parus major	234	2	19	1	0	0	0	0	0	18
Passer domesticus	4	1	0	0	0	0	0	0	0	0
Phoenicurus ochruros	1	0	0	0	0	0	0	0	0	0
Phoenicurus phoenicurus	10	0	0	0	1	0	0	0	0	3
Phylloscopus bonelli	1	0	0	0	0	0	0	0	0	1
Phylloscopus collybita	35	0	0	1	0	0	0	0	0	1
Phylloscopus trochilus	11	0	0	0	0	0	0	0	0	1
Picus viridis	1	0	0	0	0	0	0	0	0	0
Poecile montanus	5	0	0	0	0	0	0	0	0	0
Poecile palustris	6	0	1	0	0	0	0	0	0	1
Prunella modularis	398	0	0	0	0	0	0	0	0	18
Pyrrhula pyrrhula	87	0	0	0	0	0	0	0	0	9
Saxicola torquata	17	1	0	0	1	0	0	0	0	2
Sitta europaea	9	0	0	0	0	0	0	0	0	0
Sturnus vulgaris	8	0	0	0	0	0	0	0	0	0
Sylvia atricapilla	292	3	0	0	3	0	0	0	0	21
Sylvia borin	8	0	0	0	0	0	0	0	0	2
Sylvia communis	70	1	0	0	0	0	0	0	0	3
Sylvia curruca	7	0	0	0	0	0	0	0	0	0
Sylvia undata	7	0	0	0	0	0	0	0	0	0
Troglodytes troglodytes	50	0	0	0	0	0	0	0	0	5
Turdus merula	454	6	0	0	1	0	0	0	0	38
Turdus philomelos	155	1	0	0	0	0	0	0	0	9
Turdus pilaris	1	0	0	0	0	0	0	0	0	0
Turdus viscivorus	1	0	0	0	0	0	0	0	0	2

Table S2. Number of TBP-positive engorged tick larvae per pathogen and host bird species.

**Pathogen Species Bird Species**	***A. pha***	***B. afz***	***Bbss***	***B. gar***	***Bor* spp.**	***B. tur***	***B. val***	***B. miy***	***Bab* spp.**	***B. ven***	***E. can***	***Ehr* spp.**	**Close to *E. chaff***	***R. aes***	***R. hel***	***R. slo***	***Rick* spp.**
Acrocephalus palustris	0	0	0	1	0	0	0	0	0	0	0	0	0	0	0	0	0
Acrocephalus scirpaceus	0	0	0	0	0	0	0	0	0	0	0	0	0	0	0	0	0
Aegithalos caudatus	0	0	0	0	0	0	0	0	0	0	0	0	0	0	0	0	0
Anthus trivialis	1	0	0	5	0	0	1	0	0	0	0	0	0	0	1	0	0
Aquila chrysaetos	1	0	0	0	0	0	0	0	0	0	0	0	0	0	0	0	1
Carduelis chloris	1	0	0	0	0	0	0	0	0	0	0	0	0	0	0	0	0
Coccothraustes coccothraustes	0	0	0	0	0	0	0	0	0	0	0	0	0	0	0	0	1
Emberiza cirlus	0	0	0	0	0	0	0	0	0	0	0	0	0	0	0	0	0
Emberiza schoeniclus	0	0	0	0	0	0	0	0	0	0	0	0	0	0	0	0	0
Erithacus rubecula	18	2	0	3	0	0	0	2	9	1	0	3	4	2	7	0	9
Fringilla coelebs	2	0	0	0	0	0	0	0	0	0	0	0	0	0	1	0	0
Garrulus glandarius	3	0	0	0	0	0	0	0	2	0	0	0	0	0	2	0	0
Hippolais polyglotta	0	0	0	0	0	0	0	0	0	0	0	0	0	0	0	0	0
Lanius collurio	0	0	0	0	0	0	0	0	0	0	0	0	0	0	0	0	0
Limosa limosa	0	0	0	0	0	0	0	0	0	0	0	0	0	0	0	0	0
Luscinia megarhynchos	2	0	0	2	0	0	0	1	0	0	0	0	0	0	5	0	1
Cyanistes caeruleus	0	0	0	0	0	0	0	0	0	0	0	0	0	0	0	5	1
Lophophanes cristatus	0	0	0	0	0	0	0	0	0	0	0	0	0	0	0	0	0
Parus major	12	0	0	4	0	1	0	1	4	0	0	0	0	1	3	5	3
Poecile montanus	0	0	0	0	0	0	0	0	0	0	0	0	0	0	0	0	0
Phoenicurus phoenicurus	0	0	0	0	0	0	0	0	0	0	0	0	0	0	0	0	0
Phylloscopus collybita	0	0	0	1	0	0	0	0	0	2	0	0	0	0	1	0	1
Phylloscopus trochilus	0	0	0	0	0	0	0	0	0	0	0	0	0	0	0	0	0
Prunella modularis	0	0	0	2	0	0	0	1	3	0	1	2	1	0	0	0	0
Pyrrhula pyrrhula	0	0	0	0	0	0	0	0	0	0	0	0	0	0	0	0	0
Saxicola torquata	0	0	0	0	0	0	0	0	0	0	0	0	0	0	0	0	0
Sitta europaea	0	0	0	0	0	0	0	0	0	0	0	0	0	0	0	0	0
Sylvia atricapilla	3	0	0	2	0	0	0	1	0	0	0	0	0	1	10	0	1
Sylvia borin	0	0	0	0	0	0	0	0	0	0	0	0	0	0	0	0	0

Sylvia communis	1	0	1	0	0	0	0	2	0	0	0	0	0	0	1	0	0
Sylvia curruca	0	0	0	1	0	0	0	0	0	0	0	0	0	0	0	0	0
Sylvia undata	0	0	0	1	0	0	0	0	0	0	0	0	0	0	0	0	0
Troglodytes troglodytes	1	0	0	8	0	0	1	0	0	0	0	0	0	0	2	0	1
Turdus merula	14	0	0	55	0	0	12	0	2	0	0	4	0	0	9	0	1
Turdus philomelos	4	0	0	15	1	3	6	3	2	0	0	0	0	0	3	0	3
Turdus pilaris	0	0	1	0	0	0	0	0	0	0	0	0	0	0	0	0	0
**Total**	63	2	2	100	1	4	20	11	22	3	1	9	5	4	45	10	23

A. pha = *A. phagocytophilum*, *B. afz* = *B. afzelii*, *Bbss* = *Borrelia burgdorferi* sensu stricto, *B. gar* = *B. garinii*, *Bor* spp. = *Borrelia* spp., *B. tur* = *B. turdi*, *B. val*= *B. valaisiana*, *B. miy* = *B. miyamotoi*, *Bab spp.* = *Babesia* spp., *B. ven* = *B. venatorum*, *E. can* = *E. canis*, *Ehr* spp. = *Ehrlichia* spp., Close to *E. chaff* = Close to *E. chaffeensis*, *R. aes* = *R. aeschlimannii*, *R. hel* = *R. helvetica*, *R. slo* = *R. slovaca*, *Rick.* spp. = *Rickettsia* spp.

Table S3**.** Accession numbers of submitted sequences.

Tick and Tick-Borne Pathogen Species	Accession Number	Percent Identity to GenBank Sequence	Comment
I. ricinus	ON387755	100%	Compared to the sequence KF197132.1 in GenBank that came from a host-seeking female collected in Italy
I. frontalis	ON387754	99.8%	Compared to the sequence KU170492.1 in GenBank that came from a tick collected in Hungary
H. concinna	ON303650	100%	Compared to the sequence KY364906.1 in GenBank that came from a tick collected in China
H. punctata	ON387756	100%	Compared to the sequence NC_062064.1 in GenBank that came from an adult tick collected on cattle in China
B. venatorum	ON171470	99.6%	Compared to the sequence MG344777.1 in GenBank that came from the blood of *Capreolus capreolus* in Czech Republic
B. afzelii	ON310820)	100%	Compared to the sequence MT007941.1in GenBank that came from a host-seeking tick collected in Russia
Borrelia burgdorferi sensu stricto	ON310821	100%	Compared to the sequence MF150052.1 in GenBank that came from a host seeking *I. ricinus* collected in Poland
B. garinii	ON310822	99.5%	Compared to the sequence KU672556.1 in GenBank that came from a host-seeking *I. persulcatus* collected in Russia
B. turdi	ON310824	98.9%	Compared to the sequence D85071.1 in GenBank
B. valaisiana	ON310823	99.3%	Compared to the sequence MF150077.1 in GenBank that came from a host-seeking *I. ricinus* collected in Poland
B. miyamotoi	ON398526	100%	Compared to the sequence LC540659.1 in GenBank that came from an *I. ricinus* collected in Russia
E. canis	-	100%	Compared to the sequence MT066094.1 in GenBank that came from a *Rhipicephalus sanguineus* collected on a *Canis lupus familiaris* in Egypt
Close to *E. chaffeensis*	ON390894	99.2%	Compared to the sequence NR_074500.2 in GenBank
R. aeschlimannii	ON310825	100%	Compared to the sequence KU961544.1 in GenBank that came from a host-seeking *H. marginatum*

Note: *E. canis* sequence was too small to be submitted on GenBank. Here is the sequence:
CCACCTACGTGCCCTTTACGCCCAATAATTCCGAACAACGCTTGCCCCCTCCGTATTACCGCGGCTGCTGGCACAGAGTT
TGCCGGGACTTCTTCTATAGGTACCGTCATTATCTTCCCT

Table S4. Percentage of co-infected larvae (calculated on the total of engorged larvae, n = 1,106).

Pathogen Species	Percentage of Infected Larvae (n)
Anaplasma* spp. + *Bbsl	**1% (11)**
A. phagocytophilum + *B. garinii*	0.7% (8)
A. phagocytophilum + *B. valaisiana*	0.3% (3)
Rickettsia* spp. + *Bbsl	**0.9% (10)**
B. garinii + *R. helvetica*	0.5% (6)
B. garinii + *R. slovaca*	0.1% (1)
B. valaisiana + *R. helvetica*	0.1% (1)
B. garinii + *Rickettsia spp.*	0.1% (1)
B. turdi + *Rickettsia* spp.	0.1% (1)
***Anaplasma* spp. + *Rickettsia* spp.**	**0.4% (4)**
A. phagocytophilum + *R. helvetica*	0.3% (3)
A. phagocytophilum + *R. aeschlimannii*	0.1% (1)
***Babesia* spp. + *Rickettsia* spp.**	**0.3% (3)**
B. venatorum + *Rickettsia* spp.	0.1% (1)
Babesia spp. + *R. aeschlimannii*	0.1% (1)
Babesia spp. + *Rickettsia spp.*	0.1% (1)
B. miyamotoi* + *Bbsl	**0.2% (2)**
B. miyamotoi + *B. garinii*	0.2% (2)
***Anaplasma* spp. + *Ehrlichia* spp.**	**0.1% (1)**
A. phagocytophilum + Ehrlichia spp.	0.1% (1)
Babesia* spp. + *Bbsl	**0.1% (1)**
Babesia spp. + *B. garinii*	0.1% (1)
***Anaplasma* spp. + *Babesia* spp. + *Rickettsia* spp**	**0.2% (2)**
A. phagocytophilum + *Babesia* spp. + *Rickettsia* spp.	0.1% (1)
A. phagocytophilum + *Babesia* spp. + *R. aeschlimannii*	0.1% (1)
Anaplasma* spp.+ *Ehrlichia* spp. + *Bbsl	**0.1% (1)**
A. phagocytophilum + *B. valaisiana* + *Ehrlichia* spp.	0.1% (1)
Anaplasma* spp.+ *Rickettsia* spp. + *Bbsl	**0.1% (1)**
A. phagocytophilum + *B. garinii* + *R. helvetica*	0.1% (1)

Babesia* spp. + *Ehrlichia* spp. + *Bbsl	**0.1% (1)**
Babesia spp. + *B. garinii* + *Ehrlichia* spp.	0.1% (1)
Anaplasma* spp. + *B. miyamotoi* + *Bbsl	**0.1% (1)**
A. phagocytophilum + *B. valaisiana* + *B. miyamotoi*	0.1% (1)

Bbsl: *Borrelia burgdorferi* sensu lato

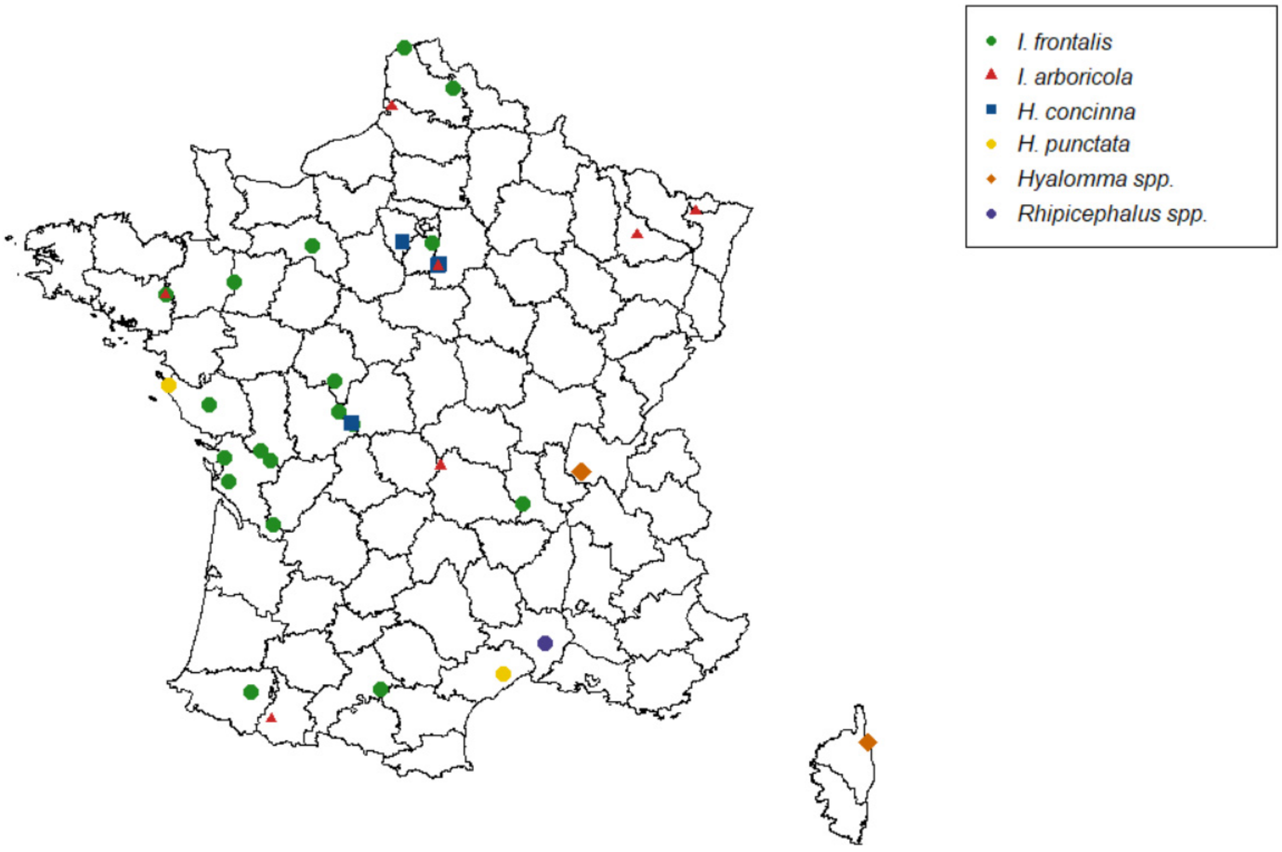

Figure S1. Geographical distribution of tick species other than *I. ricinus* (*I. frontalis, I. arboricola, H. concinna, H. punctata, Hyalomma* spp. and *Rhipicephalus* spp.) collected from breeding birds in 2019 and 2020. Green dots and red triangle represent the sites where *I. frontalis* and I. arboricola were collected respectively. Blue squares and yellow dots represent the sites where *H. concinna* and *H. punctata* ticks were collected respectively. Orange diamond represent the sites where *Hyalomma* spp. ticks were collected and purple dots represent the sites where *Rhipicephalus* spp. ticks were collected.

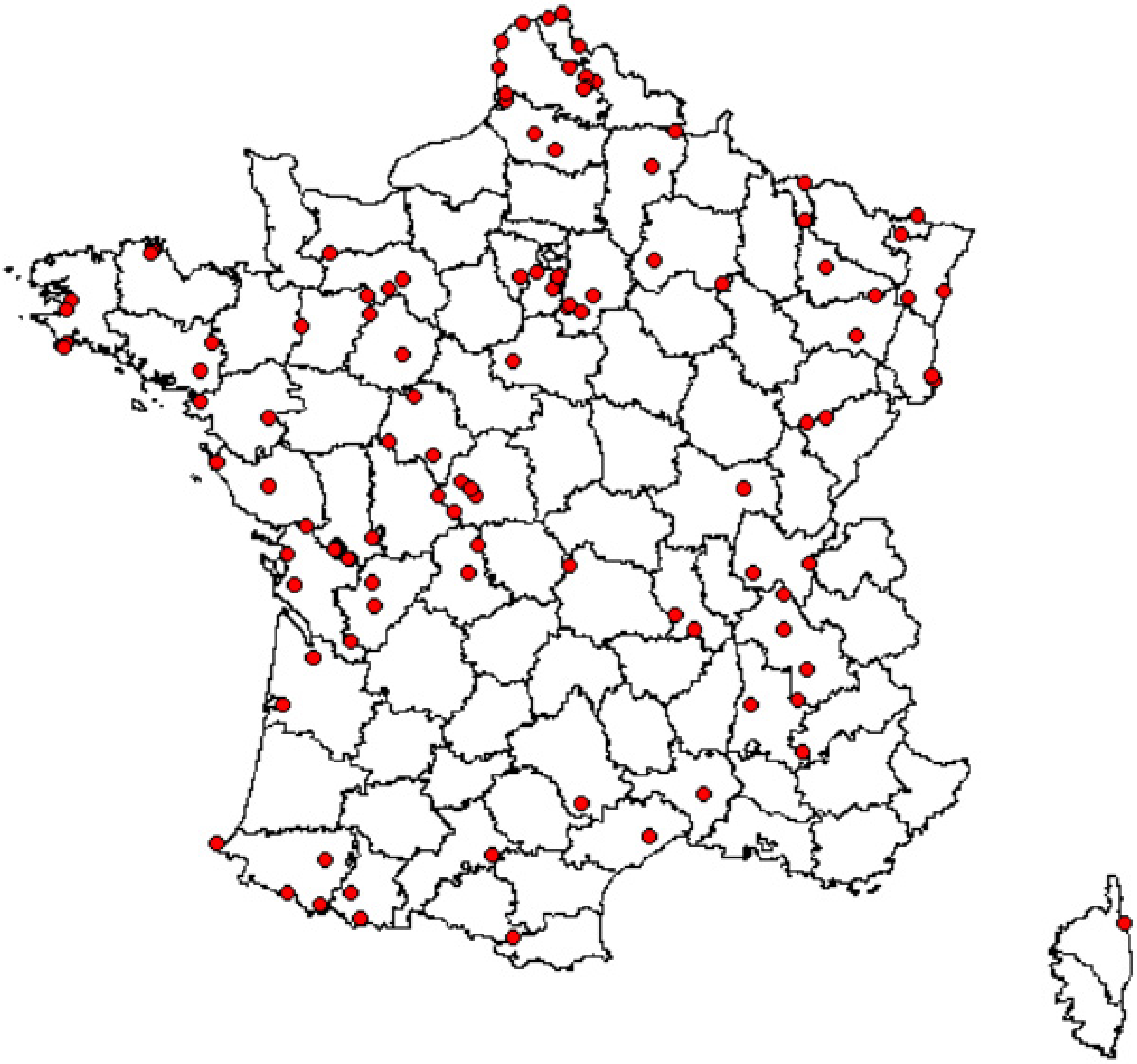

Figure S2. Capture sites for breeding birds in France in 2019-2020.
